# Prevalence of ocular complications of sickle cell disease in children seen at a tertiary health facility in Southern Ghana

**DOI:** 10.1093/jscdis/yoag009

**Published:** 2026-02-16

**Authors:** Imoro Zeba Braimah, Vera M Beyuo, Catherine Segbefia, Andrew D Campbell, Charles Antwi-Boasiako, Vera A Essuman

**Affiliations:** Ophthalmology Unit, Department of Surgery, University of Ghana Medical School, University of Ghana, Accra, Greater Accra Region GP 4236, Ghana; Eye Department, Korle Bu Teaching Hospital, Accra, Greater Accra Region KB 77, Ghana; Eye Department, Korle Bu Teaching Hospital, Accra, Greater Accra Region KB 77, Ghana; Department of Child Health, University of Ghana Medical School, University of Ghana, Greater Accra Region GP 4236, Ghana; Division of Hematology, Department of Pediatrics, Children’s National Hospital and George Washington University School of Medicine and Health Sciences, Washington, DC 20010, United States; Department of Physiology, University of Ghana Medical School, University of Ghana, Accra, Greater Accra Region GP 4236, Ghana; School of Nursing, College of Health Professions and Sciences, University of Wisconsin-Milwaukee, Milwaukee, WI 53201-0413, United States; Ophthalmology Unit, Department of Surgery, University of Ghana Medical School, University of Ghana, Accra, Greater Accra Region GP 4236, Ghana; Eye Department, Korle Bu Teaching Hospital, Accra, Greater Accra Region KB 77, Ghana

**Keywords:** sickle cell disease, proliferative sickle cell retinopathy, children, Ghana

## Abstract

**Objectives:**

To determine the spectrum of ocular complications of sickle cell disease (SCD) and age of onset of proliferative sickle cell retinopathy (PSR) in children attending the pediatric clinic at a tertiary hospital in southern Ghana.

**Methods:**

A cross-sectional study of all children with SCD in steady state, ages 2 to 16 years. Demographic and clinical data were recorded using predesigned forms. The ocular manifestations of SCD were classified as either proliferative or non-proliferative based on the presence or absence of neovascularization, respectively.

**Results:**

Included in this study were 294 children, (53.4% males), mean age 9.2 ± 3.7 years and majority had HbSS-199 (67.9%) and HbSC-74 (25.3%) genotypes. Comma-shaped conjunctiva vessels were the commonest anterior segment manifestation occurring in 85% of children. Non-proliferative posterior segment signs included: venous tortuosity 69 (23.5%), salmon-patch hemorrhages 4 (1.4%), iridescent spots 38 (12.9%), and black sunburst 24 (8.2%). Prevalence of PSR of any stage among children with HbSS was 2.5% (5/199) and 8.1% (6/74) among those with HbSC (*P = .*0366). The earliest age PSR was detected in this cohort was 8 years and for PSR stage 3, 13 years. No child was blind from eye complications of SCD or any other cause.

**Conclusion:**

Comma-shaped conjunctiva vessels were the commonest anterior segment manifestation of SCD, and retinal venous tortuosity was the most common posterior segment manifestation. Proliferative retinopathy was more common in HbSC genotype.

## Teaser

Sickle cell disease (SCD) can affect the eyes, with manifestations ranging from subtle changes to vision-threatening complications. Find out more on how SCD affects the eyes in children from this study conducted in Ghana, a country with a high prevalence of SCD.

## INTRODUCTION

Sickle cell disease (SCD) is the commonest inherited hemoglobinopathy in the world with the greatest burden in West Africa.^1^^,^^2^ Approximately 80% of all children born with SCD are in Sub-Saharan Africa.^2^ In Ghana 2% of new-born are diagnosed with SCD and many thousands are living with the disease.^3^

In SCD, there is polymerization of abnormal hemoglobin (Hb) of red blood cells (RBCs) under reduced oxygenation, leading to sickling of RBCs in different tissue vasculature and resultant complications.^1^^,^^4^^,^^5^ Sickle cell retinopathy (SCR) is characterized by microvascular occlusions that affect the peripheral retinal vasculature resulting in ischemia and the development of proliferative sickle cell retinopathy (PSR).^6^^,^^7^ PSR may result in vitreous hemorrhage, fibrosis, and contraction leading to tractional retinal detachment which are the most common causes of visual loss.^8^

The spectrum of ocular complications of SCD in the Ghanaian pediatric population and the age of onset of PSR is unknown.^9^ Various studies in other populations have observed the onset of PSR to be between the ages of 8 and 10 years for patients with the SC genotype, and 16 years for the SS genotype.^10–12^ Furthermore, the prevalence of PSR increases with age with a peak between 15 and 24 years in those with HbSC, and between 20 and 30 years in those with HbSS genotypes.^13^^,^^14^ In this study, we aimed to determine the spectrum of ocular complications of SCD and the earliest age at which PSR is detected in Ghanaian children attending the pediatric sickle cell clinic at a tertiary hospital in southern Ghana.

## MATERIALS AND METHODS

This cross-sectional study was conducted at the pediatric sickle cell clinic of a tertiary hospital in southern Ghana from July 2017 to April 2018. This study formed part of the “Determining the Sickle Cell Phenotype within the Ghanaian Sickle Cell Disease Population” sub-study of the Consortium for the Advancement of Sickle Cell Research (CASIRE): Sickle Cell Renal Disease Cohort Study. Ethical approval was obtained from the Ethical and Protocol Review Committee of the College of Health Sciences, University of Ghana [MS-Et/M.11—P4.11/2012-2013] and the research adhered to the tenets of the Declaration of Helsinki on human subjects and data protection act of Ghana (Act 843, 2012). We report on the ophthalmic aspect of the study.

All children diagnosed with SCD aged between 2 and 16 years, who were in steady state without infections, pain episodes, emergency room visit or hospitalization, in the 2 weeks prior to recruitment, and whose parents or guardians consented to participate in this study, with assent from those aged 8 years and above, were included.

Demographic and clinical data were recorded using a predesigned medical history questionnaire. A detailed ocular examination was done. This included visual acuity assessment using test appropriate for age (Sheridan Gardiner for ages 2 to 4 years and Snellen’s/logarithm of minimum angle of resolution [logMAR] charts for those aged 5 years and above). The visual acuity was further categorized as normal, mild, moderate, severe vision reduction (or visual impairment), and blindness as categorized in the ICD-11 classification.^15^ Anterior segment examination was done using a handheld slit lamp (Zeiss HSO 10 Hand slit lamp and ophthalmoscope illuminator H, Carl Zeiss Meditec AG, Jena, Germany) or binocular indirect ophthalmoscope (Keeler UK) with a 20D or 15D lens to examine in detail the conjunctiva, cornea, anterior chamber, iris, and pupil. Intraocular pressures were checked using Icare TA01i tonometer (Finland) and Neurologic examination including cranial nerves 1 to 12 was done. Dilated fundus examination using Guttae tropicamide 1% and Guttae phenylephrine 2.5% and a binocular indirect ophthalmoscope (Keeler UK) with 20D and 28D lenses.

*Study definition*: The ocular manifestation of SCD was classified as either proliferative or non-proliferative based on the presence or absence of neovascularization. Classification of proliferative sickle retinopathy in this study was based on the 5 stages of retinopathy defined by Goldberg.^16^

Stage I—peripheral arteriolar occlusion,

Stage II—peripheral arteriovenous anastomosis,

Stage III—pre-retinal neovascularization and fibrous proliferation,

Stage IV—vitreous hemorrhages due to proliferative retinopathy,

Stage V—tractional retinal detachment due to mechanical traction from fibrotic sea fans.

Non-proliferative signs of SCD recorded in this study included anterior segment changes such as Comma-shaped conjunctival vessel segments and iris atrophy as previously described.^17^^,^^18^ Non-proliferative retinal signs of SCD were based on the signs described in previous studies such as retinal venous tortuosity, salmon-patch hemorrhages, iridescent spots, black sunburst spot, and angioid streaks.^6^^,^^19^^,^^20^

### Treatment of cases identified

Participants were treated depending on clinical findings and complications, and those requiring laser photocoagulation and retinal detachment surgery were also treated. These treatments were as per medication and other treatment modalities used and prescribed routinely in Ghana as per Ghana health service (GHS)/Teaching Hospitals treatment policies as well as those of the National Health Insurance Scheme (NHIS).

### Follow-up of patients

Follow-up visits for participants with ocular complications were scheduled depending on the type and severity of the findings.

### Statistical analysis

Data was entered into Microsoft Access 2010 and data analysis was done using the Statistical Package for Social Sciences (SPSS version 20.0). Descriptive statistics was used for data analysis and the results summarized in percentages, proportions and means ± standard deviations. The results were presented in tables as appropriate. Fischer’s exact test and chi-square test were used to determine the association between dependent and independent variables. The significance level was set at .05.

## RESULTS

Two hundred ninety-four children (157 [53.4%] males and 137 [46.6%] females) were included in this study. The mean age ± standard deviation (SD) of the participant at eye examination was 9.2 ± 3.7 years (median, 9 years, range, 2-16.75 years). Their sickle cell hemoglobin genotype status included HbSS-199 (67.9%), HbSC-74 (25.3%), HbAS 2 (0.7%), HbSF 13 (4.4%), HbSD 2 (0.7%) and HbS/Beta thalassemia 3 (1%). Previous systemic complications (number) obtained from the medical records of the participants are summarized in Table 1. Two children had previous blood transfusion during treatment of priapism and abnormally high transcranial Doppler ultrasound values.

**Table 1.** Previous systemic complications in children with sickle cell disease in Korle Bu Teaching Hospital, Ghana.

Systemic complication	Number (%)
Acute chest syndrome	9 (3.1)
Vaso-occlusive pain episodes	14 (4.8)
Severe anemia	9 (3.1)
Osteomyelitis	4 (1.4)
Pneumonia	3 (1.0)
Priapism	2 (0.7)
Others	
Congestive cardiac failure	2 (0.7)
Cerebrovascular accident	2 (0.7)
Sickle cell hepatopathy	1 (0.3)
Nephritic syndrome	1 (0.3)

The mean log MAR best corrected visual acuities were 0.06 ± 0.13 and 0.05 ± 0.12 in the right and left eyes, respectively. Two hundred forty-one children (82.0%) had unaided Snellen Visual acuity between 6/5 and 6/9 in their right eyes and 240 (81.6%) in the left eyes. Thirteen (4.4%) had unaided visual acuity between 6/18 and 6/60 (moderate visual impairment) in their right eyes and 7 (2.4%) in the left eyes. No child had visual acuity worse than 6/60.

Ocular adnexal manifestations in the children are summarized in Table 2. The mean intraocular pressures were 15.7 ± 3.1 mmHg (range 8-25, median 16 mmHg) and 15.5 ± 3.3 mmHg (range 8-26, median 16) in the right and left eyes, respectively.

**Table 2.** Ocular adnexal manifestation of children with sickle cell disease in Korle Bu Teaching Hospital, Ghana.

Ocular manifestations	Right eye: *N* (%)	Left eye: *N* (%)
Adnexal/ocular surface		
** Ptosis**	1 (0.3)	1 (0.3)
** Blepharitis**	4 (1.4)	4 (1.4)
** Chalazion**	2 (0.7)	1 (0.3)
** Allergic conjunctivitis**	33 (11.2)	33 (11.2)
** Coma-shaped conjunctival vessels**	233 (85.3)	233 (85.3)
** Conjunctival naevus**	2 (0.7)	2 (0.7)
** Jaundice**	8 (2.7)	8 (2.7)
Anterior segment		
** Iris pigment epithelial cyst**	–	1 (0.3)
** Lisch nodules**	1 (0.3)	1 (0.3)
** Iris naevus**	1 (0.3)	–
** Pseudogerontoxon**	1 (0.3)	1 (0.3)

The mean vertical optic cup to disc ratio was 0.3 ± 0.15 (range 0.0-0.8, median 0.3) and 0.3 ± 0.1 (range 0.0-0.8, median 0.3) in the right and left eyes, respectively. The following non-proliferative posterior segment signs of sickle cell retinopathy were observed in the children: venous tortuosity (69, 23.5%), salmon patch hemorrhages (4, 1.4%), iridescent spots (38, 12.9%) and black sunburst spots (24, 8.2%). Eleven (3.7%) children had proliferative sickle cell retinopathy (PSR) of any stage. The prevalence of PSR of any stage among children with HbSS was 2.5% (5/199) and among those with HbSC was 8.1% (6/74), *P = .*0366. The earliest age at which PSR of any stage was detected was 8 years and for PSR stage 3, 13 years. Ten (3.4%) children had stage 1 PSR, 7 (2.4%) had stage 2 PSR, and 5 (1.7%) children had stage 3 PSR. The sea fan neovascularization (stage 3 PSR) was raised in 2 out of the 5 children. PSR stage 3 2/199 (1.0%) in HbSS and 3/74 (4.1%) in HbSC, *P = .*095. The posterior segment signs including the types of PSR observed in the right and left eyes of the children are summarized in Table 3. None of the children had PSR stage 4 or 5.

**Table 3.** Demographic and clinical characteristics, and association between sickle cell genotypes SS and SC among children in Korle Bu Teaching Hospital, Ghana.

Demographic/clinical characteristics and posterior segment manifestations	Sickle cell genotypes			*P* value
	SS genotype *N* (%) 199 (72.9)	SC genotype *N* (%) 74 (27.1)	Total *N* (%) 273 (100%)	
**Age**				.599
**0-5 years**	39 (19.6)	18 (24.3)	57 (20.9)	
** 6-10 years**	76 (38.2)	29 (39.2)	105 (38.4)	
** 11-16 years**	84 (42.2)	27 (36.5)	111 (40.7)	
**Sex**				.497
** Male**	103 (51.6)	42 (56.8)	145 (53.1)	
** Female**	96 (48.2)	32 (43.2)	128 (46.9)	
** Total**	199 (100.0)	74 (100.0)	273 (100.0)	
**Comma shaped conjunctival vessels**	181 (91.0)	52 (70.3)	233 (85.3)	.001^a^
**Venous tortuosity**	50 (25.1)	15 (20.3)	65 (23.8)	.429
**Salmon patch hemorrhage**	4 (2.0)	–	4 (1.5)	.577
**Iridescent spots**	31 (15.6)	6 (8.1)	37 (13.6)	.162
**Black sunburst pigmentation**	20 (10.1)	2 (2.7)	22 (8.1)	.048^a^
**PSR: PSR1^b^**	5 (2.5)	6 (8.1)	11 (3.7)	.037^a^
** PSR2**	3 (1.5)	4 (5.4)	7 (2.6)	.089
** PSR3**	2 (1.0)	3 (4.1)	5 (1.8)	.125
**PSR3: Flat**	2 (1.0)	1 (1.4)	3 (1.1)	1.000
** Raised**	–	2 (2.7)	2 (0.7)	.073
**Retinal detachment**	–	1 (1.4)	1 (0.4)	.271

a Statistically significant association.

b PSR, proliferative sickle cell retinopathy stage.

Rhegmatogenous retinal detachment was observed in the right eye of a 9-year-old child with retinal tear posterior to the equator which was successfully treated with scleral buckle surgery using radial buckle (silicon sponge 7.5mm × 5.5mm) and 14-year-old child with retinal hole and localized inferior retinal detachment in the right eye was treated with laser photocoagulation.

## DISCUSSION

This study reports the spectrum of ocular manifestation of sickle cell complications and the age of onset of proliferative retinopathy in Ghanaian children aged between 2 years and 16 years. Non-proliferative signs were more prevalent than proliferative signs. Comma-shaped conjunctival vessels was the commonest anterior segment manifestation occurring in 85% of the children and retinal venous tortuosity was the most common posterior segment manifestation occurring in 24% of the children. Clinically significant proliferative retinopathy (Goldberg stage 3) was rare and was found in 1.8% of the children. The earliest age at which proliferative retinopathy (Goldberg stage 3) was detected was 13 years and no child was blind from eye complications of SCD or any other cause.

Comma-shaped conjunctival vessel abnormality is a common feature of sickle cell hemoglobin SS and SC disease.^9^^,^^17^^,^^21^^,^^22^ They are seemingly isolated conjunctival vascular segments with one or both ends often comma-shaped and are thought to occur from vascular stasis.^17^ Abiose and Lesi^21^ reported that 81.3% of 91 children with HbSS below age 15 years attending the pediatric sickle cell anemia clinic in Lagos University Teaching Hospital in 1977 had conjunctival vessel abnormalities, and these were seen in 91% of the children above 10yrs of age. However, Osafo-Kwaako et al.^9^ observed the presence of comma sign in 63% of patients attending the adult sickle cell clinic in Korle-Bu Teaching Hospital, Ghana. The variation in the prevalence of comma-shaped conjunctival sign may be attributed to differences in the technique of the conjunctival examination, genotype of SCD, previous blood transfusion and age of the patient.^9^^,^^17^^,^^22^ Comma-shaped conjunctival sign is best recognized by examining the bulbar conjunctiva near the lower fornix using a slit lamp biomicroscope.^17^ It has been observed to accumulate and increase in severity with age particularly in HB SS genotype.^22^ In this study we observed the comma-shaped conjunctival sign was more common in the HB SS genotype and a trend of increasing prevalence with age.

There are several retinal manifestations of SCD. The non-proliferative retinal signs have been observed to be more common in children with the HB SS genotype compared to the HB SC genotype.^9^^,^^19^ This is in contrast with observation in adolescents and adults, where proliferative retinopathy (Goldberg stage 3) is significantly more common in the HB SC disease compared to HB SS disease.^19^ In a cross-sectional study of 96 children of average age 6 years 3 months, Talbot et al.^19^ observed that retinal changes were more common in children with SS disease than SC. Discrete retinal patches similar to schisis cavities resulting from intraretinal hemorrhage was observed in 37% children with SS compared to 24% of children with SC, and peripheral arteriolar closure (Goldberg stage 1) was observed in 24% of the children with SS compared to 16% of children with SC although none of the children developed proliferative retinopathy (Goldberg stage 3).^19^ In a study of 54 children with HB SC disease aged between 2 and 15 years, Condon and Serjeant^20^ observed, 32% of the children had retinal venous tortuosity, 17% had iridescent glistening spots, and 7% had black sunburst. Similarly, this current study observed retinal venous tortuosity in 20%, iridescent spots in 8% and black sunburst in 3% of children with HB SC disease. The rate of non-proliferative retinal findings in children with SS disease in this study is similar to the observation of Abiose and Lesi^21^ which was reported 4 decades ago. Abiose and Lesi^21^ observed the following non-proliferative signs in children with HB SS disease, retinal venous tortuosity (27%), black sunburst (11%), refractile retinal deposits/iridescent spots (12%), and retinal hemorrhage (salmon patch) 4%. A recent cross-sectional study by Oluleye et al.^23^ in Ibadan observed 14% of children with SCD had retinal venous tortuosity in contrast to the 24% observed in this study. This current study is of a larger sample size and the children were, on average, about 2 years older compared to those studied by Oluleye et al.^23^

Proliferative retinopathy is more common in HB SC genotype compared to HB SS genotype.^13^^,^^14^ It has been found to be rare in the first decade of life; increases with age^10^^,^^13^ and peaks at age 15-24 years in males with HB SC disease, 20-39 years in females with HB SC disease and 25-39 years in HB SS disease.^13^ Condon and Serjeant^20^ observed that 17 out of 54 (31.5%) with HB SC disease had peripheral arteriolar occlusion (Goldberg stage 1 PSR) and 6 (11%) had sea fan neovascularization (Goldberg stage 3 PSR). Abiose and Lesi^21^ observed peripheral arteriolar occlusion in 5% and neovascularization in 1% of children with HB SS genotype. The new vessel was found in an 8-year-old child.^21^ In a longitudinal cohort study of 307 SS and 166 SC subjects, Downes et al.^10^ reported that the earliest age of onset of PSR was 8 years for SC patients and 16 years for SS patients. In addition, a retrospective longitudinal study of 263 children under 18 years of age by Gill and Lam^11^ found the mean age of onset of PSR to be 12.8 years (range 9-18 years) with PSR occurring at an earlier age in the SC genotype, 9 years compared to SS genotype, 16 years and a prevalence of PSR of 2.7%; with PSR predominantly occurring in the SC genotype (8.2%) compared to the SS genotype (0.6%). The prevalence of PSR (Goldberg stage 3) in this current study is 1.8%, occurring more frequently in the SC genotype (4.1%) compared to the SS genotype (1%) and 13 years was the earliest age at which it was detected. The age of onset of PSR (Goldberg stage 3) in the current study could not be accurately determined because of the study design (cross-sectional study) and the new vessels observed had undergone regression leaving flat or raised fibrovascular membranes. Furthermore, the prevalence of PSR may be affected by the introduction of hydroxyurea in the management of SCD in some of our patients. Low fetal hemoglobin (hemoglobin) has been observed to be a risk factor of PSR, and hydroxyurea increases hemoglobin F levels in patients with SCD.^24^

Although children with SCD develop proliferative retinopathy (Goldberg stage 3) which is sight threatening, blindness from this complication is rare. Downes et al.^10^ observed spontaneous regression in 32% of affected eye, little progression in the remainder and 2 developed visual loss from tractional retinal detachment. In the study reported by Gill and Lam,^11^ none of the children had permanent loss of vision related to sickle cell retinopathy. No child had visual acuity worse than 6/60 in our study and one child with rhegmatogenous retinal detachment with macular involvement had improvement in visual acuity from 6/60 to 6/18 following scleral buckle surgery and a second child with localized inferior peripheral retinal detachment was treated with laser photocoagulation. It should be noted that RD in individuals with SCD may not only be from PSR but also from retinal tears and holes.^25^ Screening for sight threatening sickle retinopathy (Goldberg stage 3) has been recommended to begin at age 10 years particularly in those with the SC genotype by some investigators.^10–12^ There is the need for caution in recommending screening for PSR at this early age in our setting due to low prevalence of the disease, favorable natural history of PSR in children and limited resources. A multicenter, longitudinal evaluation of children with SCD in Ghana would be useful in determining the natural history of PSR in Ghana as well as guide future national policy and management and may influence management of children with SCD in Sub-Saharan Africa as well. Meanwhile, there is the need for increasing awareness creation of PSR among pediatricians treating children with SCD so they can refer such children for periodic evaluation.

Limitations of this study include the cross-sectional study design and hence the earliest age of onset of proliferative SCD could not be established with certainty and loss of some data due to inability to examine some of the children enrolled. In addition, it is possible some early stages of PSR were not diagnosed due to absence of adjunctive imaging such as ultrawide field fluorescein angiography.^26^ However, to the best of our knowledge, it is the first study in Ghana to describe the current ocular manifestations of SCD in the pediatric population.

## CONCLUSION

Comma-shaped conjunctival segments were the commonest anterior segment manifestation of SCD and retinal venous tortuosity is the most common posterior segment manifestation. Proliferative retinopathy was more common in the SC genotype, and no child was blind from complications of sickle cell retinopathy.
